# Biomass augmentation through thermochemical pretreatments greatly enhances digestion of switchgrass by *Clostridium thermocellum*

**DOI:** 10.1186/s13068-018-1216-7

**Published:** 2018-08-04

**Authors:** Ninad Kothari, Evert K. Holwerda, Charles M. Cai, Rajeev Kumar, Charles E. Wyman

**Affiliations:** 1 Dept. of Chemical and Environmental Engineering, Bourns College of Engineering, University of California Riverside (UCR), Riverside, CA USA; 2 Center for Environmental Research and Technology (CE-CERT), Bourns College of Engineering, University of California Riverside, Riverside, CA USA; 3 Thayer School of Engineering, Dartmouth College, Hanover, NH USA; 4 BioEnergy Science Center (BESC), Oak Ridge National Laboratory (ORNL), Oak Ridge, TN USA; 5 Center for Bioenergy Innovation (CBI), Oak Ridge National Laboratory (ORNL), Oak Ridge, TN USA

**Keywords:** Bioethanol, *Clostridium thermocellum*, Consolidated bioprocessing, Pretreatment, Switchgrass

## Abstract

**Background:**

The thermophilic anaerobic bacterium *Clostridium thermocellum* is a multifunctional ethanol producer, capable of both saccharification and fermentation, that is central to the consolidated bioprocessing (CBP) approach of converting lignocellulosic biomass to ethanol without external enzyme supplementation. Although CBP organisms have evolved efficient machinery for biomass deconstruction, achieving complete solubilization requires targeted approaches, such as pretreatment, to prepare recalcitrant biomass feedstocks for further biological digestion. Here, differences between how *C. thermocellum* and fungal cellulases respond to senescent switchgrass prepared by four different pretreatment techniques revealed relationships between biomass substrate composition and its digestion by the two biological approaches.

**Results:**

Alamo switchgrass was pretreated using hydrothermal, dilute acid, dilute alkali, and co-solvent-enhanced lignocellulosic fractionation (CELF) pretreatments to produce solids with varying glucan, xylan, and lignin compositions. *C. thermocellum* achieved highest sugar release and metabolite production from de-lignified switchgrass prepared by CELF and dilute alkali pretreatments demonstrating greater resilience to the presence of hemicellulose sugars than fungal enzymes. 100% glucan solubilization and glucan plus xylan release from switchgrass were achieved using the CELF–CBP combination. Lower glucan solubilization and metabolite production by *C. thermocellum* was observed on solids prepared by dilute acid and hydrothermal pretreatments with higher xylan removal from switchgrass than lignin removal. Further, *C. thermocellum* (2% by volume inoculum) showed ~ 48% glucan solubilization compared to < 10% through fungal enzymatic hydrolysis (15 and 65 mg protein/g glucan loadings) of unpretreated switchgrass indicating the effectiveness of *C. thermocellum*’s cellulosome. Overall, *C. thermocellum* performed equivalent to 65 and better than 15 mg protein/g glucan fungal enzymatic hydrolysis on all substrates except CELF-pretreated substrates. CELF pretreatments of switchgrass produced solids that were highly digestible regardless of whether *C. thermocellum* or fungal enzymes were chosen.

**Conclusions:**

The unparalleled comprehensive nature of this work with a comparison of four pretreatment and two biological digestion techniques provides a strong platform for future integration of pretreatment with CBP. Lignin removal had a more positive impact on biological digestion of switchgrass than xylan removal from the biomass. However, the impact of switchgrass structural properties, including cellulose, hemicellulose, and lignin characterization, would provide a better understanding of lignocellulose deconstruction.

**Electronic supplementary material:**

The online version of this article (10.1186/s13068-018-1216-7) contains supplementary material, which is available to authorized users.

## Background

Fuel ethanol derived from abundant lignocellulosic biomass has the highest potential to alleviate the dependence on fossil petroleum in the transportation sector while also dramatically reducing associated greenhouse gas emissions [1, 2]. The conversion of lignocellulosic feedstocks into ethanol currently requires a combination of size reduction, thermochemical pretreatment, fungal enzyme production, enzymatic hydrolysis, sugar fermentation, and product recovery [3, 4]. The high capital and operating costs of these processes, especially that of production and purification of large doses of cellulolytic enzymes from *Trichoderma reesei* required to obtain sufficiently high sugar yields, challenge the economic competitiveness of cellulosic ethanol [5]. Thus, intense research is required to reduce the amount of fungal enzymes needed or eliminate their use to decrease processing costs.

Consolidated bioprocessing (CBP) is an improved method that reduces enzyme-related costs using cellulolytic microorganisms that have the ability to produce their own enzyme consortium to simultaneously hydrolyze biomass polysaccharides and ferment the released sugars into ethanol and other desirable bioproducts [6, 7]. *Clostridium thermocellum* is a particularly promising CBP cellulolytic microorganism [3, 6, 8] that is capable of nearly completely digesting cellulose materials such as Avicel on its own [9]. In contrast, even though high, only 48% of the cellulose in natural Alamo switchgrass was solubilized by *C. thermocellum* as reported in this work. Thus, cellulose accessibility to digestion in a complex substrate such as biomass remains the primary barrier to economic production of cellulosic ethanol and methods to overcome biomass recalcitrance to breakdown remain a subject of intense study [4, 10, 11]. For biological systems, recalcitrance for a certain type of biomass material has been attributed to the presence of lignin and hemicellulose in the biomass interfering with cellulose macro-accessibility, the physical availability of cellulose to enzymatic saccharification [12–15]. In fact, an increase in accessibility of the substrate to saccharolytic enzymes of *C. thermocellum* via ball milling during cotreatment of switchgrass has been shown to increase solubilization [16]. Various biomass pretreatment techniques have also been developed that can also prepare lignocellulosic biomass for further conversion to ethanol by targeting removal of hemicelluloses or lignin to ensure greater cellulose accessibility [11, 17, 18].

Substrate adaptive changes in *C. thermocellum* cellulosomes reported in literature suggest that the organism adjusts the proportion of different activities in the cellulosome, such as endoglucanases, exoglucanases, xylanases, and pectinases, in response to substrate features [19]. However, a comprehensive evaluation of CBP performance on different substrates and targeted integration of CBP with biomass augmentation technologies has not been done. A significant amount of CBP literature is focused on at most one or two instead of a range of pretreatment methods and/or conditions for maximization of either polysaccharide solubilization or ethanol production [20–23]. On the other hand, a number of past studies have reported results from application of various pretreatment types and pretreatment conditions followed by use of fungal enzymes for further digestion [18, 24–26]. Other, more focused studies attempted to elucidate the impact of lignin or hemicellulose removal on enzymatic hydrolysis by fungal enzymes [27–30]. Along those lines, here we aim to systematically show the effect of biomass composition, specifically the presence of lignin and hemicellulose (represented by xylan), on sugar solubilization and metabolite production by *C. thermocellum*.

## Results and discussion

### Different pretreatments of switchgrass create compositionally distinct substrates

Alamo switchgrass was selected as a fast-growing energy crop rich in both pentose and hexose sugars to serve as the model feedstock for this study [31]. In addition to unpretreated switchgrass, compositionally distinct materials were prepared by subjecting switchgrass to four different pretreatment methods: hydrothermal, dilute acid, dilute alkali, and co-solvent-enhanced lignocellulosic fractionation (CELF). These pretreatments were chosen based on their abilities to distinctively solubilize hemicellulose sugars (quantified as xylan) or lignin (measured as Klason-lignin or acid-insoluble lignin) or both from unpretreated biomass [32, 33]. CELF pretreatment was recently developed to employ tetrahydrofuran (THF) as a miscible co-solvent to water used in combination with dilute acid to enhance biomass delignification and depolymerization [32]. Pretreatment conditions were varied over a range of reaction temperatures and times to help determine conditions at which maximum glucan plus xylan release from pretreatment (Stage 1) in combination with *C. thermocellum* CBP (Stage 2) was observed for each pretreatment type. Sugar recovery was tracked starting from both glucan and xylan in unpretreated biomass as established elsewhere for integration of pretreatments with fungal enzymatic hydrolysis [34].

**Fig. 1:**
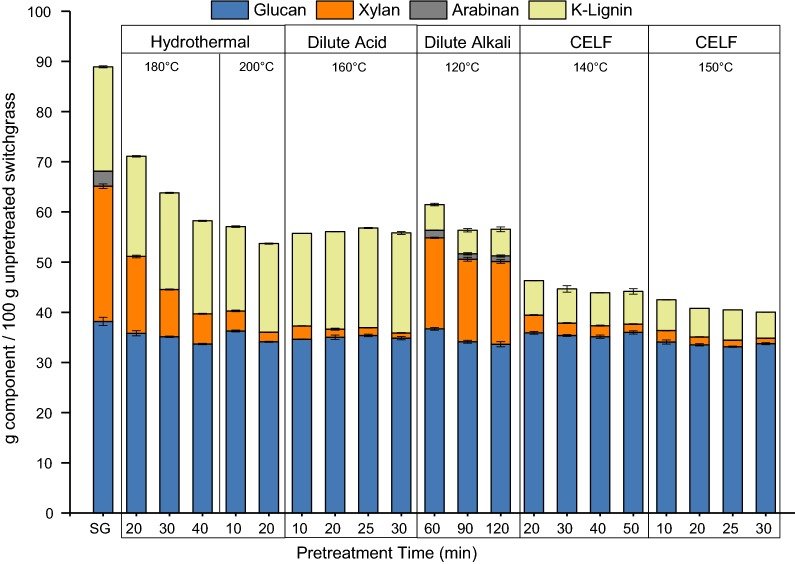
Tracking fate of components of switchgrass in solids before and after hydrothermal, dilute acid, dilute alkali, and co-solvent enhanced lignocellulosic fractionation (CELF) pretreatments adjusted to a basis of 100 g of initial unpretreated switchgrass (SG). Pretreatments were performed at 10% solids loading (80 g switchgrass on a dry basis) with a total reaction mass of 800 g

Total mass of glucan, xylan, and lignin found in unpretreated switchgrass and pretreated solids was tracked starting from 100 g equivalent of the unpretreated material as shown in Fig. 1 to reveal both relative compositions of each component and mass changes after pretreatment. Overall, the solubilization of glucan during pretreatment was minimal for all pretreatment types increasing slightly with longer reaction times. For CELF pretreatment, temperature had a greater impact on glucan content in pretreated solids allowing tuning of different conditions to modify relative compositions of each component. Hydrothermal and dilute acid pretreatments achieved high xylan removal, about 44–93% and 89–96%, respectively, while only about 5–14% lignin removal was observed. Notably, dilute acid pretreatment resulted in increased lignin in pretreated solids compared to hydrothermal pretreatment associated with formation of pseudo-lignin through polysaccharide degradation, which has been shown to inhibit enzymatic digestion [35, 36]. Aggressive lignin removal was achieved by dilute alkali pretreatment, ranging from 71 to 76%, while only about 30–41% xylan removal was observed. High xylan content in dilute alkali pretreated solids is expected to influence solubilization by *C. thermocellum* and fungal enzymes differently. CELF pretreatment removed about 85–96% xylan and 67–76% lignin at pretreatment conditions applied in this study. Overall, removal of either xylan or lignin or both from switchgrass, thereby increasing macro-accessibility of cellulose to enzymes, is expected to aid further biological deconstruction of pretreated solids. Although glucan composition in solids varied across hydrothermal, dilute acid, dilute alkali, and CELF pretreatments, being 49–59, 59–60, ~ 55, 74–78%, respectively, CELF-pretreated solids contained the most glucan relative to xylan and lignin and are expected to be the most digestible. High xylan content of hydrothermal and dilute alkali solids and high lignin content of dilute acid-pretreated solids would serve to provide a wide range of compositionally distinct solids to evaluate substrate–enzyme/bacterium effects.

### Substrate composition affects *C. thermocellum* consolidated bioprocessing

**Fig. 2:**
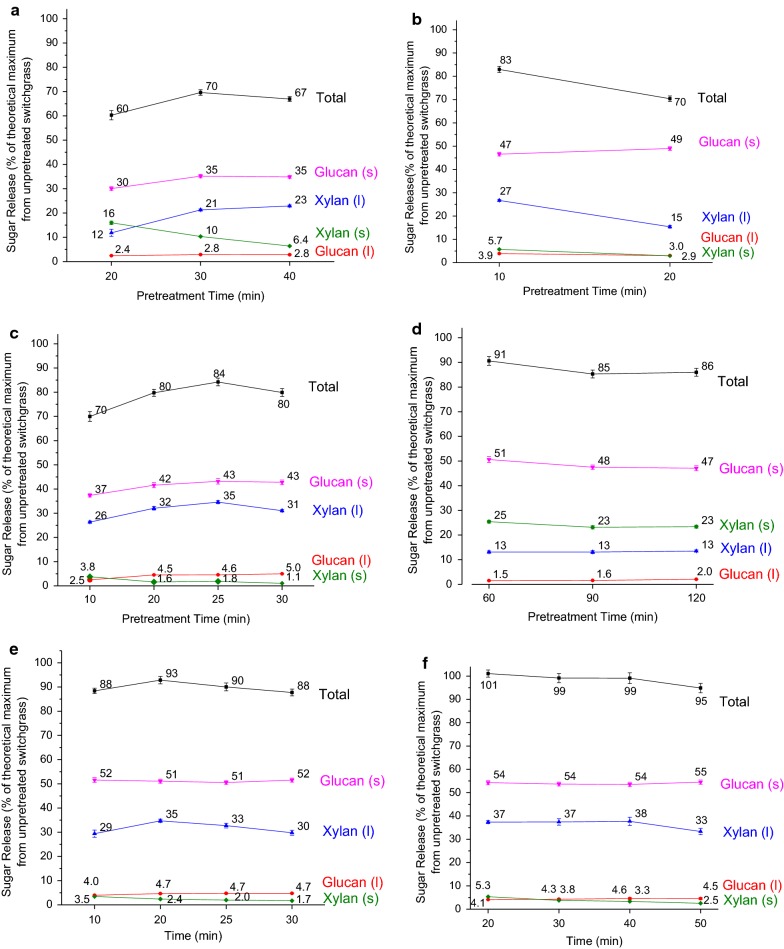
Glucan and xylan releases from Stage 1 (pretreatment; designated as “l”) and Stage 2 (7 days of *C. thermocellum* consolidated bioprocessing (CBP); designated as “s”) for **a** hydrothermal (180 °C), **b** hydrothermal (200 °C), **c** dilute acid, **d** dilute alkali, **e** co-solvent-enhanced lignocellulosic fractionation (CELF; 150 °C), and **f** CELF (140 °C) pretreatments. Pretreatments were performed at 10% solids loading (80 g switchgrass on a dry basis) with a total reaction mass of 800 g. CBP was performed at a 0.5 wt% glucan loading of all substrates with a working mass of 50 g

**Fig. 3:**
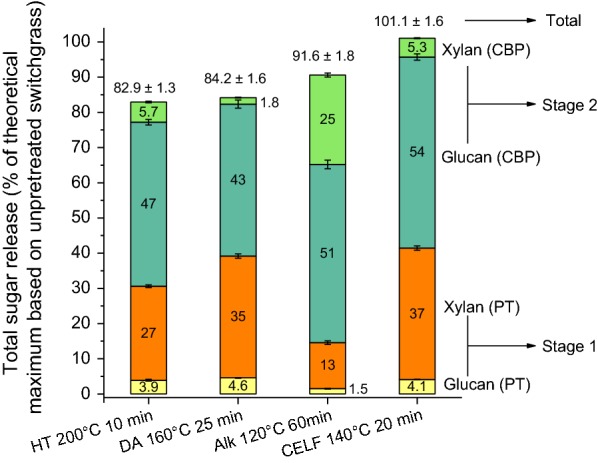
Total combined sugar (glucan plus xylan) release from Stage 1 (pretreatment, designated as “PT”) and Stage 2 (7 days of *C. thermocellum* consolidated bioprocessing, designated as “CBP”) for solids resulting from hydrothermal (HT), dilute acid (DA), dilute alkali (Alk), and co-solvent-enhanced lignocellulosic fractionation (CELF) pretreatments at conditions that gave the highest total sugar release. Pretreatments were performed at 10% solids loading (80 g switchgrass on a dry basis) with a total reaction mass of 800 g. CBP was performed at a 0.5 wt% glucan loading of all substrates with a working mass of 50 g

Pretreatment conditions were varied to determine conditions at which maximum glucan plus xylan release from pretreatment/Stage 1 (l) combined with CBP/Stage 2 (s) of switchgrass was observed for each pretreatment technology as shown in Fig. 2. Carbohydrates broken down during pretreatment were recovered in the pretreatment liquor as either monomers, oligomers, or degradation products thereof depending on pretreatment type and conditions. Pretreatment conditions for maximum sugar release critically depended on these Stage 1 sugar yields. These pretreatment conditions were harsh enough to produce biologically digestible solids but were not severe enough for degradation of sugars, especially hemicellulose sugars, released during pretreatment in the liquor. As understood from Fig. 2, the conditions for hydrothermal pretreatment at which maximum total sugar release was achieved were 200 °C for 10 min. The corresponding pretreatment conditions for dilute acid were 160 °C for 25 min, dilute alkali were 120 °C for 60 min, and CELF were 140 °C for 20 min. For the sake of clarity, Fig. 3 shows total sugar release from Stage 1 (orange/yellow) and Stage 2 (light/dark green) at these pretreatment conditions for each pretreatment technology based on glucan plus xylan present initially in unpretreated switchgrass. Although different pretreatments solubilized different amounts of sugars during Stage 1, *C. thermocellum* was highly capable of releasing the remaining sugars from the solids during Stage 2. About 100% total sugar release was achieved by CELF pretreatment of switchgrass in combination with CBP, which is the highest ever shown. Higher glucan solubilization and total sugar release observed from CELF- and dilute alkali-pretreated biomass compared to those observed from dilute acid and hydrothermal pretreatments indicate that removal of lignin from biomass by pretreatment had a greater impact on digestion by *C. thermocellum* than removal of xylan and/or lignin relocation. This is further validated by the higher Stage 2 sugar release achieved by *C. thermocellum* on delignified materials (CELF- and dilute alkali-pretreated solids) than on hydrothermal- and dilute acid-pretreated solids which are rich in lignin.

**Fig. 4:**
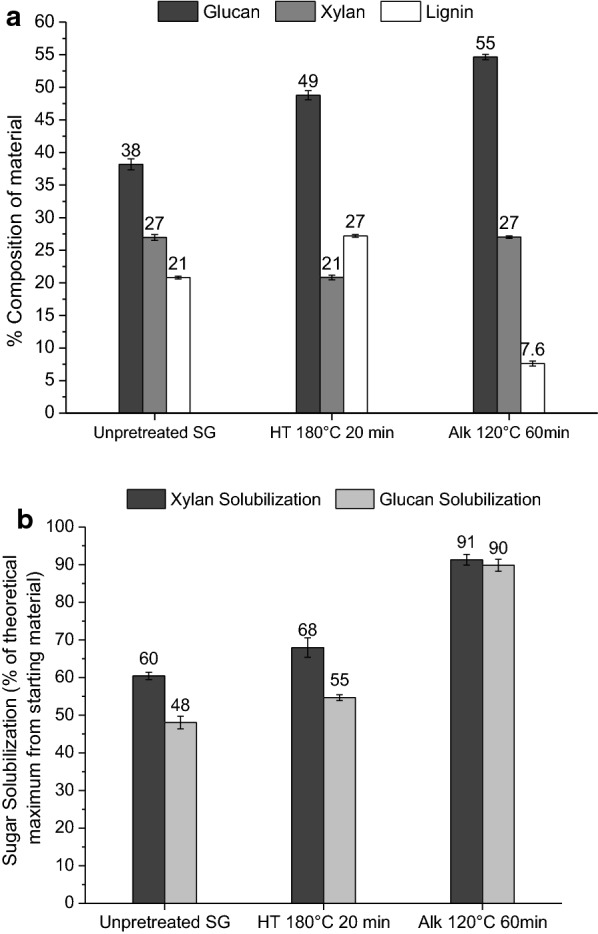
**a** % Compositions of unpretreated switchgrass (SG), hydrothermal-pretreated solids at 180 °C for 20 min, and dilute alkali-pretreated solids at 120 °C for 60 min and **b** corresponding glucan and xylan solubilizations of these materials by *C. thermocellum*

The greater effect of lignin removal on enzymatic saccharification has been attributed to increased cellulose accessibility to enzymatic digestion due to the absence of lignin and a reduction in unproductive binding of enzymes to lignin as reported for fungal enzymes [37–39]. Lignin removal from the biomass may have a similar effect on biomass deconstruction by *C. thermocellum* as well. Further, the greater effect of lignin removal on glucan solubilization by fungal enzymes has also been attributed to an increase in xylan accessibility and, therefore, digestibility due to the absence of lignin [40, 41]. This mechanism may apply to biomass deconstruction by *C. thermocellum* as well [42, 43]. Interestingly, high xylan release achieved during CBP contributed to the high Stage 2 sugar release observed on dilute alkali-pretreated solids. *C. thermocellum* was able to break down xylan even though the wild-type strain used in this work is not known to ferment xylose or xylo-oligomers [19]. Further, we decided to compare the impact of xylan removal by *C. thermocellum* on glucan solubilization by the organism from dilute alkali-pretreated solids with low lignin content (~ 8% for solids pretreated at 120 °C for 60 min) compared to hydrothermal-pretreated solids with high lignin content (~ 27% for solids pretreated at 180 °C for 20 min) as shown in Fig. 4. Both materials had high xylan contents of 21% in hydrothermal and 27% in dilute alkali pretreated solids. The result that *C. thermocellum* removed 91% xylan from dilute alkali-pretreated solids but only 68% xylan from hydrothermal-pretreated solids supports the argument that lignin removal increased xylan accessibility to *C. thermocellum*. This increased xylan digestion from dilute alkali-pretreated biomass by *C. thermocellum* may have contributed to the higher glucan solubilization of 90% compared to only 55% from hydrothermal-pretreated solids as presented in Fig. 4b. This interpretation is further supported by *C. thermocellum* being able to achieve only 48% glucan solubilization from unpretreated switchgrass with high lignin content for which the organism could only remove 60% of the xylan. Further, removal of acetyl and uronic acid substitutions from biomass hemicellulose by dilute alkali pretreatments may have also contributed to the higher cellulose and hemicellulose accessibility to digestion [4].

### Substrate composition affects *C. thermocellum* and fungal enzymes differently

**Fig. 5:**
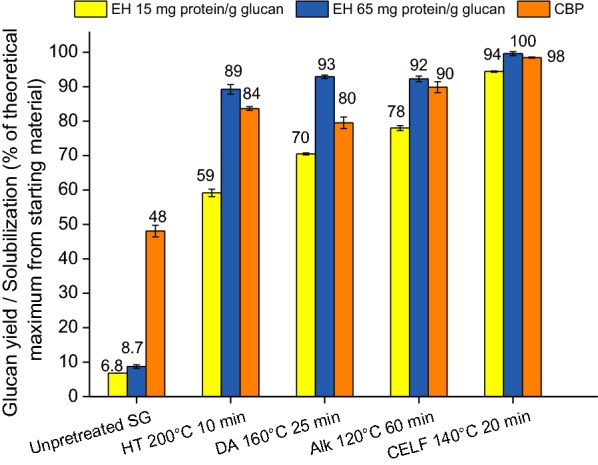
Comparison of *C. thermocellum* consolidated bioprocessing (CBP) glucan solubilizations and fungal enzyme-mediated enzymatic hydrolysis (EH) glucan yields after 7 days of each biological operation for unpretreated switchgrass (SG) and solids prepared by hydrothermal (HT), dilute acid (DA), dilute alkali (Alk), and co-solvent-enhanced lignocellulosic fractionation (CELF) pretreatments at conditions that gave the highest total sugar release. Both CBP and EH were performed at 0.5 wt% glucan loading of all substrates with a working mass of 50 g. A 2% by volume inoculum was used for *C. thermocellum* CBP and 15 and 65 mg protein/g glucan loading of Accellerase^®^ 1500 were used for EH

The deconstruction performances of different pretreatment–CBP combinations were compared to deconstruction by traditional use of fungal enzymes following the same four pretreatments of switchgrass, with results shown in Fig. 5. For the sake of clarity, only the conditions that gave the highest total sugar release from each pretreatment coupled with CBP are shown in this figure. Glucan deconstruction is measured as glucan yield in liquid for enzymatic hydrolysis and based on glucan solubilization from pretreated solids after fermentation by CBP divided by glucan initially loaded. It has been shown previously that the different methods of measuring sugar release are comparable [44]. Fungal enzymes achieved only a 7% glucan yield from unpretreated switchgrass even at a high and expensive enzyme loading of 65 mg protein/g glucan in contrast to *C. thermocellum* breaking down 48% of the glucan in unpretreated switchgrass. As a reference point, 20 mg protein/g glucan loading has been projected to cost about $1.47/gal of ethanol [5]. Although all materials were autoclaved for sterilization prior to CBP while this was not applied prior to enzymatic hydrolysis, autoclaving conditions are far too mild to increase digestibility by *C. thermocellum* as shown in Additional file 1: Fig. S1. This yield difference is an important distinction between the two biological approaches and highlights the effectiveness of the *C. thermocellum* cellulosome and/or the enzyme–microbe synergy observed during *C. thermocellum* digestions [45]. It is important to point out that the fungal enzyme system used in the current work, Accellerase^®^ 1500, is not known to contain xylanases. Better performance by CBP may be attributed to the ability of *C. thermocellum* to break down xylan in biomass to make cellulose more accessible. Others have reported synergies between xylanases and cellulases to boost hydrolysis performance on pretreated biomass by increasing cellulose accessibility either via xylan removal or increased swelling and porosity of cellulose fibers [46, 47]. In addition, xylanases may break down the lignin carbohydrate complexes to improve substrate digestibility [47].

As expected, glucan-rich material from CELF-pretreated switchgrass was the most digestible irrespective of the biological system considered. Enzymatic hydrolysis at 65 mg protein/g glucan and CBP both showed almost 100% glucan yield/solubilization from CELF-pretreated solids; however, a slightly lower glucan yield resulted at 15 mg/g glucan enzyme loading. Altogether, *C. thermocellum* always performed better than fungal enzymes did at 15 mg protein/g glucan and the same as achieved by 65 mg/g glucan enzyme loadings. The presence of xylan in the substrate may have affected enzymatic hydrolysis at the lower enzyme loadings as also validated by low glucan yield on hydrothermal compared to dilute acid-pretreated solids at that enzyme loading. *C. thermocellum*, in contrast, showed slightly higher glucan solubilization on hydrothermal than dilute acid-pretreated solids suggesting minimal impact of higher xylan present in the former on the organism. The effect of the presence of xylan in the substrate on enzymatic hydrolysis is also evident from the low glucan yield from dilute alkali-pretreated solids at the low enzyme loading compared to that by *C. thermocellum*. Even though enzymatic hydrolysis at the higher enzyme loading was not significantly impacted by varying substrate features, the high enzyme loading is expected to affect processing costs negatively. Overall, *C. thermocellum* CBP showed high glucan solubilization, especially on substrates with low lignin content.

### Lignin and xylan removal affects both solubilization and metabolite production by *C. thermocellum*

**Fig. 6:**
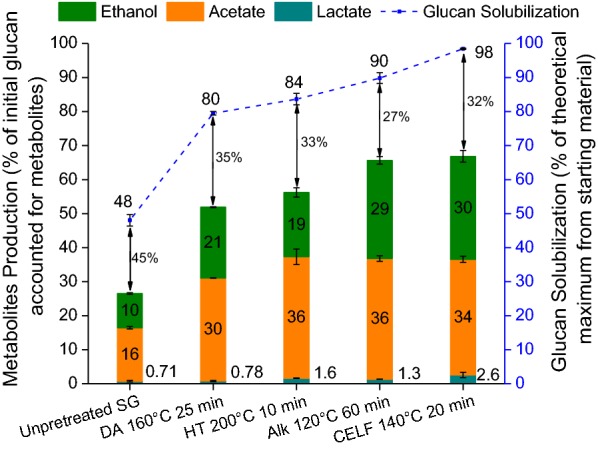
*Clostridium thermocellum* glucan solubilization and metabolite production after 7 days of fermentation on unpretreated and hydrothermal (HT), dilute acid (DA), dilute alkali (Alk), and co-solvent-enhanced lignocellulosic fractionation (CELF) pretreated switchgrass. Values on the arrows indicate the percentage of solubilized glucan that is unaccounted

The translation of glucan solubilization from biomass to production of fermentation metabolites by *C. thermocellum* is shown in Fig. 6. Metabolite production is reported as the percentage of stoichiometric amount of glucan accounted for generation of each metabolite released based on initial glucan loaded in fermentation. 67% of initial glucan in CBP was accounted for ethanol, acetic acid, and lactic acid yield by *C. thermocellum* on CELF-pretreated solids, ~ 1% higher than metabolite yield on dilute alkali, ~ 10% higher than on hydrothermal, and ~ 15% higher than on dilute acid-pretreated solids and ~ 40% higher than metabolite production on unpretreated switchgrass. Overall, total metabolite production correlated with an increase in cellulose macro-accessibility and solubilization on solids produced by all the pretreatments employed; higher polysaccharide solubilization translated into higher product yields. Although the amount of ethanol produced is greater from dilute alkali-pretreated solids than from hydrothermal-pretreated solids, the amount of acetic acid produced remains the same. This result suggests that the organism shifted its carbon flux from acetic acid to ethanol production in response to stressful conditions. Acetic acid production is preferred by *C. thermocellum* due to the generation of adenosine triphosphate (ATP) [48]. A shift in carbon flux from acetic acid and ethanol production to lactic acid on CELF-pretreated solids compared to metabolites produced on dilute alkali-pretreated solids further confirmed stressful conditions experienced by the organism. *C. thermocellum* production of lactic acid has been reported to be part of its overflow metabolism that is not essential for carbon and electron flow [9, 49]. Along with direct product inhibition, pH drop due to the formation of acetic acid and other carboxylic acids could be the primary cause of these stressful conditions.

Figure 6 also points out that 55–73% of the solubilized glucan can be attributed to major metabolites, acetic acid, ethanol, and lactic acid; while the rest is expected to have been used for cell growth and maintenance, production of enzymes, and other products [9]. Highest fraction of solubilized glucan accounted for metabolite production by *C. thermocellum* was 73% from dilute alkali-pretreated solids, suggesting that *C. thermocellum* benefits more from lignin removal than xylan removal. Although *C. thermocellum* deconstruction of CELF-pretreated solids showed the greatest glucan solubilization of 98%, about 68% of this solubilized glucan was accounted for metabolite production, 5% lower than that from dilute alkali-pretreated solids. The lower utilization of solubilized glucan on CELF-pretreated solids may perhaps be because the organism was inhibited by higher product concentrations, particularly that of acetic acid, and/or a drop in pH to non-optimal conditions.

## Conclusions

These results provide a unique picture on how compositional differences from switchgrass prepared by four different pretreatments impact subsequent deconstruction of solids by *C. thermocellum* compared to fungal enzymatic hydrolysis. Hydrothermal, dilute acid, dilute alkali, and CELF pretreatments substantially increased glucan solubilization compared to that realized from unpretreated switchgrass. Nonetheless, *C. thermocellum* was capable of achieving ~ 48% glucan solubilization on unpretreated switchgrass compared to about 7% glucan yield by fungal enzymes. The dramatic difference in deconstruction performance showed that *C. thermocellum* can break down lignocellulosic biomass more effectively than fungal enzymes. The fact that dilute alkali pretreatment increased glucan solubilization by *C. thermocellum* to ~ 90%, higher than from solids produced by either hydrothermal or dilute acid pretreatments at reasonable conditions indicated that lignin removal from switchgrass had a greater effect on total sugar release than xylan removal and/or lignin relocation. *C. thermocellum* was not adversely affected by the presence of xylan in the biomass (~ 2.5 g/L during fermentation at 5 g/L glucan loading) even though the wild-type strain of the organism is not known to ferment xylose or xylo-oligomers [19]. Such benefits of *C. thermocellum*’s complex cellulosome can be useful in selecting industrial-scale pretreatments in the future. However, *C. thermocellum* is shown to be inhibited at higher concentrations of xylose and xylo-oligomers during fermentation at higher solid loadings [50]. Nearly theoretical and rapid deconstruction of CELF-pretreated solids by *C. thermocellum* showed that biomass augmentation through thermochemical pretreatments aids biomass digestion by the organism. Further, the trends in production of metabolites, ethanol, acetic acid, and lactic acid, by *C. thermocellum* correlated well with trends in its glucan solubilization from pretreated solids. These results suggest that CELF pretreatment combined with *C. thermocellum* CBP could serve as a useful reference point against which to measure deconstruction of lignocellulosic biomass in addition to offering a promising process of converting biomass to ethanol with high yields without added enzymes. Further, it is important to consider critical differences in processing attributes and their effects on costs associated with each pretreatment. Hydrothermal pretreatments have the advantage of not requiring neutralization or conditioning because of no use of chemicals but require higher pretreatment temperature and, therefore, pressure to produce digestible solids. Temperature, as high as 200 °C, was required for hydrothermal pretreatments to achieve the same total sugar release as dilute acid pretreatments at 160 °C. However, dilute acid pretreatment requires expensive reactor materials of construction due to the corrosive nature of sulfuric acid and encounters other challenges such as, high cost of acid, challenges in mixing acid, the need for acid neutralization [4], and sugar degradation during hydrolysate conditioning prior to fermentation. Dilute alkali pretreatments have the advantage of being effective at low-temperature conditions, with only 120 °C being sufficient to be effective for this study. However, the pretreatment time is longer, ranging from 1 to 2 h in this study. Moreover, alkali is more expensive than sulfuric acid, and removal and recovery of alkali is difficult [4]. Polar aprotic solvent THF used for CELF pretreatment enhances the performance of dilute acid and can be produced sustainably from biomass [32]. Moreover, its low boiling point of ~ 66 °C makes it easy to be recovered and recycled after pretreatment. Removing THF from the liquid stream produced by CELF for recycle precipitates lignin as a solid to leave an aqueous phase rich in hemicellulose sugars. These sugars are then available for fermentation after conditioning of the liquor. However, a thorough techno-economic analysis of each pretreatment technology combined with CBP will be essential in the future in comparing different pretreatment technologies. Further, an in-depth characterization of biomass properties including cellulose, hemicellulose, and lignin characterization will provide essential insights into lignocellulosic biomass deconstruction.

## Methods

### Substrate

Five-year-old fully mature Alamo switchgrass harvested in January 2014 that had been chopped to an approximate size of ¾ inch was obtained from Genera Energy Inc. The composition of unpretreated milled Alamo switchgrass was determined to be 38.18 (± 0.8)  % glucan, 26.96 (± 0.4)  % xylan, 2.97 (± 0.05) % arabinan, and 20.8 (± 0.2)  % K-lignin. This biomass was completely mixed before dividing and transferring it into multiple gallon-sized bags that were stored in a freezer. The entire contents of each bag were knife milled by Thomas Wiley^®^ mill (Model 4, Thomas Scientific, Swedesboro NJ) equipped with a 1-mm sieve. The resulting milled biomass was mixed thoroughly before each use.

### Pretreatment

Hydrothermal pretreatments were performed at 180 °C for 20, 30, and 40 min and 200 °C for 10 and 20 min. Dilute acid pretreatments were done at 160 °C for 10, 20, 25, and 30 min, whereas, dilute alkali pretreatments were done at 120 °C for 60, 90, and 120 min. CELF pretreatments were performed at 140 °C for 20, 30, 40, and 50 min and 150 °C for 10, 20, 25, and 30 min. Dilute acid and CELF pretreatments used 0.5 wt% sulfuric acid, whereas dilute alkali pretreatments were with 1 wt% sodium hydroxide. CELF pretreatment employed THF as co-solvent at a 1:1 volume ratio with water/dilute sulfuric acid solution. These pretreatment conditions were based on prior work and reported in the literature, [32, 51–54] and because the results followed trends consistent with prior results in most cases, the pretreatments were performed once without replicates. A 10 wt% solids loading (80 g on a dry basis) was fed to all pretreatments based on a total of 800 g reaction mass. Before pretreatment, biomass was soaked overnight with the other ingredients at room temperature. The biomass for CELF, however, was soaked at 4 °C to minimize solvent evaporation. A 1-L Hastelloy Parr reactor (236HC series, Parr Instruments Co., Maoline, IL) equipped with a double-stacked pitch blade impeller rotating at 200 rpm was used for all pretreatments. A 4-kW fluidized sand bath (Model SBL-2D, Techne, Princeton, NJ) maintained the pretreatment temperature within ± 2 °C. The reactor temperature was measured by a K-type thermocouple probe (CAIN-18G-18, Omega Engineering Co., Stamford, CT, USA). The heat-up time of the reactor contents from 30 °C to the desired temperature was usually between 3 and 4 min. At the completion of the target pretreatment time, the reactor was lowered into a room temperature water bath to cool its contents to ~ 80 °C in about 2 min. The contents of the reactor were then vacuum filtered at room temperature using a glass fiber filter paper, and a sample of the pretreatment liquor was taken for analysis. The solids were thoroughly rinsed with room temperature deionized water to remove any soluble sugars, degradation products, acid/alkali, and solvents. Since the co-solvent mixture had a different density than pure water, density of the CELF pretreatment liquor was determined by weighing 25 mL of the liquor in a volumetric flask immediately after the reaction. The composition of the liquor was measured by HPLC following the standard NREL Laboratory Analytical Procedure “Determination of sugars, byproducts, and degradation products in liquid fraction process samples” [55]. The sugar yields reported in the pretreatment liquor included both monomers and oligomers. The rinsed solids were subjected to further biological deconstruction using either *C. thermocellum* or fungal enzymes.

### *Clostridium thermocellum* consolidated bioprocessing

The DSM 1313 wild-type strain of *C. thermocellum* employed for all experiments were kindly provided by Dr. Lee Lynd at Dartmouth College, Hanover NH. A stock culture was grown in a 500-mL anaerobic media bottle (Chemglass Life Sciences, Vineland NJ) and aseptically transferred to 5-mL serum vials for storage at − 80 °C. A 2% by volume inoculum of these stock cultures were used to prepare the seed cultures grown with a 5 g/L glucan loading of Avicel^®^ PH-101 (Sigma Aldrich, St. Louis, MO) in a 50 mL working volume for 8–9 h in media for thermophilic clostridia (MTC) without trace minerals (Table 1). The pellet nitrogen content and metabolite production by *C. thermocellum* grown in this manner in a working volume of 200 mL is shown in Additional file 2: Fig. S2. Pellet nitrogen content was analyzed according to methods published in literature [9, 56].

**Table 1:** Media for thermophilic clostridia (MTC) for *C. thermocellum* CBP

Solution	Component	Reactor concentration (g/L)	Stock concentration (g/L)
A	MOPS (buffer)	10	100
B	Citric acid potassium salt [C_6_H_5_K_3_O_7_·H_2_O]	2	50
	Citric acid monohydrate	1.25	31.25
	Na_2_SO_4_	1	25
	KH_2_PO_4_	1	25
	NaHCO_3_	2.5	62.5
C	NH_4_Cl	1.5	75
D	MgCl_2_·6H_2_O	1	50
	CaCl_2_·2H_2_O	0.2	10
	FeCl_2_·4H_2_O	0.2	5
	l-Cysteine hydrochloride monohydrate	1	50
E	Pyridoxamine dihydrochloride	0.02	1
	p-Aminobenzoic acid	0.004	0.2
	d-Biotin	0.002	0.1
	Vitamin B12	0.002	0.1

MOPS, 3-(*N*-morpholino)propanesulfonic acid (p*K*_a_ 7.20)

The seed culture was stored overnight in a refrigerator for about the same time for each experiment before inoculation the next day. The different media solutions were prepared separately and purged with nitrogen. All solutions except the vitamin solution were sterilized by autoclaving, whereas the vitamin solution was sterilized by passing it through 28-mm diameter polyethersulfone (PES) syringe filters with 0.2-µm pores (Corning^®^ Life Sciences, Tewksbury MA). CBP was performed in 125-mL bottles (Wheaton, Millville NJ) at a 0.5 wt% glucan loading of unpretreated or pretreated biomass in triplicates at a working mass of 50 g. Bottles containing biomass and water were purged with repeated 45-s application of vacuum and 14 psi nitrogen over a total of 27–30 min and then sterilized by autoclaving at 121 °C for 35 min. The bottles were incubated at 60 °C at a shaking speed of 180 rpm in a Multitron Orbital Shaker (Infors HT, Laurel MD) after the injection of all medium solutions and 2% by volume inoculum. Insoluble solids left after CBP were recovered and rinsed thoroughly. Compositional analysis was performed on these CBP residues to determine carbohydrates left in the solids from which solubilization could be calculated. The solids were sampled in triplicates after 7 days of fermentation to determine carbohydrate solubilization. This approach of measuring solubilization was followed because sugars released in solution are immediately used by *C. thermocellum* for fermentation to metabolites and are not left in solution as they would be for fungal enzymatic hydrolysis. The fermentation liquor was also analyzed after 7 days of fermentation to determine production of ethanol, lactic acid, and acetic acid. The liquor samples were passed through 28-mm diameter polyethersulfone (PES) syringe filters with 0.2-µm pores (Corning^®^ Life Sciences, Tewksbury, MA) into 1.5 mL Simport^®^ microcentrifuge tubes (Spectrum^®^ Chemical Manufacturing Corporation, New Brunswick, NJ). These tubes were centrifuged at 15,000 rpm for 10 min and the supernatant was analyzed by HPLC.

### Enzymatic saccharification

Unpretreated and pretreated switchgrass solids were enzymatically hydrolyzed at a loading of 0.5 wt% glucan and working mass of 50 g in 125-mL Erlenmeyer flasks in triplicates following the NREL Laboratory Analytical Procedure “Enzymatic Saccharification of Lignocellulosic Biomass” [57]. Accellerase^®^ 1500 cellulase (DuPont Industrial Biosciences, Palo Alto CA) was used at 15 and 65 mg/g glucan protein loadings for all experiments, with the loadings based on the amount of glucan in unpretreated switchgrass as described elsewhere so as to not penalize a pretreatment for releasing more sugars before enzymatic saccharification [54, 58]. The BCA protein content of Accellerase^®^ 1500 was reported elsewhere to be 82 mg/mL [59]. The flasks were incubated at 50 °C and 150 rpm in a Multitron Orbital Shaker (Infors HT, Laurel MD) and allowed to equilibrate before adding the enzyme solution. 1 mL representative samples including the insoluble substrate and liquor were collected from each flask after 4, 24 h, and every 24-h period thereafter for a total of 7 days or 168 h in 1.5 mL Simport^®^ microcentrifuge tubes (Spectrum^®^ Chemical Manufacturing Corporation, New Brunswick, NJ). These tubes were centrifuged at 15,000 rpm for 10 min and the supernatant was analyzed by HPLC.

### Compositional analysis of solids

Prior to analysis, unpretreated and pretreated solids were dried for 2 days to constant moisture content in a 40 °C incubator oven. CBP residues were bone dried in a 60 °C oven. The compositions of unpretreated and rinsed pretreated switchgrass and CBP residues were determined in triplicates according to the standard NREL Laboratory Analytical Procedure “Determination of Structural Carbohydrates and Lignin in Lignocellulosic Biomass” [60]. If the amount of material was insufficient to meet the NREL specified amount, the amounts of ingredients were modified proportionately. Glucan, xylan, arabinan, Klason-lignin (K-lignin), and ash were measured in this manner, with K-lignin accounting for all the acid-insoluble lignin in the biomass.

### Analytical procedures

Analysis of all the liquid samples was by a Waters Alliance e2695 HPLC system (Waters Co., Milford MA) equipped with a Bio-Rad Aminex HPX-87H column and a Waters 2414 refractive index detector. 5 mM sulfuric acid was the eluent at a flow of 0.6 mL/min. Integration of the chromatograms was by the Empower™ 2 software package. All experiments and analysis were in triplicates, unless otherwise specified. Error bars were calculated as the standard deviation for the triplicates, unless otherwise specified. Even though the pretreatments were only performed once the compositional analysis for all pretreated solids and pretreatment liquor was done in triplicates. Error for total sugar release was calculated based on a combination of standard deviation of glucan and xylan solubilization in CBP, glucan and xylan yields in pretreatment liquor, and glucan and xylan composition of pretreated solids following statistical rules for combining standard deviation.

### Calculations

Sugar yield, conversion, and release were expressed in terms of the polymeric form of the sugar throughout this paper with anhydrous correction factors converting monomeric sugar concentrations measured by HPLC to the corresponding polymer carbohydrate. Thus, the amount of glucose measured via HPLC can be converted to the equivalent amount of glucan by multiplying the glucose amount by 0.9, while the factor for translating xylose to xylan and arabinose to arabinan is 0.88. The glucan release from the biomass for CBP is measured in terms of glucan solubilization and that for enzymatic saccharification is measured as glucan yield as described in the main text and calculations shown below. Stage 1 sugar release refers to sugars captured in the pretreatment liquor, whereas, the Stage 2 sugar release refers to sugar solubilized by *C. thermocellum* from pretreated solids; both measurements are based on the initial amount of glucan plus xylan in unpretreated switchgrass. The term ‘sugar release’ includes both glucan and xylan in the calculations unless specified otherwise. Stage 1 and Stage 2 sugar releases combined are termed total sugar release. All of these sugar release calculations are done based on a dry mass basis. Detailed calculations are as follows:

1. $$\begin{aligned} &\text{Mass of }\left( {\text{dry}} \right)\text{ biomass loaded in Parr reactor for pretreatment }\left( \text{g} \right) \\ & = \left( {{\% }\,{\text{Solids}}\,\,\text{loading }\,\,\text{fraction}} \right) \times \left( {\text{Total reaction mass }\left( \text{g} \right)} \right) \\ \end{aligned}$$
2. $$\begin{aligned} &\text{Mass of }\left( {\text{wet}} \right)\text{ biomass loaded in Parr reactor for pretreatment }\left( \text{g} \right) \\ & = \frac{{\left( {{\% }\,\text{Solids loading}} \right) \times \left( {\text{Total reaction mass }\left( \text{g} \right)} \right)}}{{\left( {\text{100} - {\% }\,\text{moisture }\,\text{content}} \right)}} \\ \end{aligned}$$
3. $$\begin{aligned} & \text{Mass of total liquid to be loaded for pretreatment }\left( \text{g} \right)\text{ } \\ & = \text{Total reaction }\,\text{mass }\left( \text{g} \right) - \text{mass of}\,\text{ }\left( {\text{wet}} \right)\text{biomass loaded}\left( \text{g} \right) \\ \end{aligned}$$
4. $$\begin{aligned} & \text{Mass of liquid present before pretreatment }\left(\text{ g} \right)\text{ } \\ & =\text{ Total reaction mass }\left( \text{g} \right)- \text{mass of}\,\,\left( {\text{dry}} \right)\text{biomass loaded}\left( \text{g} \right) \\ \end{aligned}$$
5. $$\text{Acid/alkali loading }\left( \text{g} \right) = \frac{{\left( {\text{Mass of liquid present before pretreatment}} \right){ \times }\left( {{\% }\,\text{acid loading}} \right)}}{{\left( {{\% }\,\,\text{Concentration of acid solution}} \right)}}$$
6. $${\text{Pretreatment}}\;{\% }\,\text{ solids}\,\text{ yield}\,= \frac{{\text{Mass of dry solids recovered after pretreatment }\left( \text{g} \right)}}{{\text{Mass of }\,\,\left( {\text{dry}} \right)\text{biomass loaded before pretreatment }\left( \text{g} \right)}} \times \text{100}$$
7. $$\begin{aligned} & \text{Mass of liquid after pretreatment}\left ( \text{g} \right)\text{ } \\ & = \text{ Total reaction mass }\left( \text{g} \right) - \text{Mass of dry solids recovered after pretreatment}\left( \text{g} \right) \\ \end{aligned}$$
8. $$\text{Volume of liquid after pretreatment }\left( \text{L} \right) = \frac{{\text{Mass of liquid after pretreatment}\,\text{ }\left( \text{g} \right)}}{{\text{Density of liquid after pretreatment (g/L)}}}$$
  (density of liquid measured for CELF pretreatment and assumed 1 g/mL for aqueous pretreatments)
9. $$\begin{aligned} {\%}\,\text{Stage 1 glucan}\,\text{ or}\,\text{ xylan}\,\,\text{release} &= \text{Concentration of sugar monomer in liquid after pretreatment}\,\text{ }\left( {\text{g/L}} \right) & \\ & {\kern 1pt} \times \,\frac{{\text{Volume of liquid after pretreatment}\,\text{ }\left( \text{L} \right) \times \text{Anhydrous factor}}}{{\text{Mass of glucan plus xylan in solids before pretreatment }\left( \text{g} \right)}}{ \times 100} \\ & \\ \end{aligned}$$
10. $$\begin{aligned} {\text{CBP \%}}\,\,\text{sugar}\,\text{solubilization } &= \text{Mass of sugar polymer in solids before CBP }\left( \text{g} \right) \\ & - \frac{{\left( {{\% }\,{\text{Sugar}}\text{ }\,\,\text{polymer}\,\text{in}\,\text{solids}\,\text{after CBP} \times \text{Mass of dry solids after CBP }\left( \text{g} \right)} \right)}}{{\text{Mass of sugar polymer in solids before CBP }\left( \text{g} \right)}}{ \times 100} \\ \end{aligned}$$
11. $$\begin{aligned} & \text{Enzyme loading in enzymatic hydrolysis, mg protein/g glucan in unpretreated biomass} & \\ & \text{ = mg Protein/g}\,\,\text{glucan loaded} \times \frac{{\text{Mass of glucan in }\,\left( {\text{dry}} \right)\text{biomass before pretreatment}}}{{\text{Mass of glucan in dry solids after pretreatment}}} \\ \end{aligned}$$
12. $$\begin{aligned} \text{Enzymatic}\,\text{ hydrolysis}\,{ \% }\,\text{ sugar}\,\text{yield}& = \text{Concentration}\,\text{of}\,\text{sugar}\,\text{monomer}\,\text{measured}\,\text{in}\,\text{liquid(g/L)} \\ & \quad \times \frac{{\text{(Working volume }\left( \text{L} \right) \times \text{Anhydrous factor}}}{{\text{Mass of sugar polymer before hydrolysis }\left( \text{g} \right)}} \times \text{100} \\ \end{aligned}$$
13. $$\begin{aligned} & {\% }\,\text{Stage 2}\,\text{glucan}\,\text{or}\,\text{xylan}\;{\text{release}}\,= { \% }\;{\text{Sugar}}\,\text{yield}\,\text{or}\,\text{solubilzation} \times {\% }\,{\text{Sugar}}\,\text{polymer}\,\text{in}\,\text{pretreated}\,\text{solids} \\ & \times \frac{{\text{Mass of dry solids recovered after pretreatment }\left( \text{g} \right)}}{{\text{Mass of glucan plus xylan in solids before pretreatment }\left( \text{g} \right)}} \times \text{100} \\ \end{aligned}$$
14. $$\begin{aligned} {\text{\% Total}}\,\;\text{sugar release } = & \,{\% }{\text{ Stage 1}}\text{ }\,\text{glucan release} \\ & { +\; \% }{\text{ Stage 1 }}\text{xylan release} \\ & { +\; \% }{\text{ Stage 2 }}{ \text{glucan release + \% }}{\text{Stage 2 }}\text{xylan release} \\ \end{aligned}$$
15. $$\begin{aligned} & \text{CBP metabolites yield }\left( {\text{g metabolites starting with 100 g glucan}} \right)\text{ } \\ & = \frac{{\left( {\text{Metabolites concentration in liquid(g/L)} \times \text{Working volume in flask }\left( \text{L} \right)} \right)}}{{\text{Mass of initial glucan loaded for CBP }\left( \text{g} \right)}} \times \text{100} \\ \end{aligned}$$
16. $$\begin{aligned} & {\text{CBP metabolites yield}}\left( {\% \;{\text{of initial glucan accounted for metabolites}}} \right) \\ & \frac{{{\text{CBP metabolites production}}\left( {{\text{g metabolite starting with}} 100\; {\text{glucan}}} \right) \times {\text{Anhydrous factor}}}}{{{\text{Stoichometric factor}} \times {\text{Molar mass ratio}}}} \\ \end{aligned}$$
17. $$\begin{aligned} & \text{Stoichiometric factor }\left( {\text{for a balanced glucose to metabolite reaction}} \right) \\ & =\frac{{\text{Stoichiometric coefficient of metabolite}}}{{\text{Stiochiometric coefficient of glucose}}} \\ \end{aligned}$$
18. $$\text{Molar mass ratio}=\frac{{\text{Molar mass of metabolite}}}{{\text{Molar mass of glucose}}}$$
19. $$\begin{aligned} & \text{CBP metabolites yield }\left( {{\text{\% of solubilized glucan accounted}}\,{\text{for}}\,{\text{metabolites}}} \right) \\ & =\frac{{\text{CBP metabolites production }\left( {{\% }\,\text{of}\,\text{initial}\,\text{glucan}\,\text{accounted for metabolites}} \right)}}{{\text{CBP glucan solubilization}}} \times \text{100} \\ \end{aligned}$$
20. $$\begin{aligned}{\text{\% Solubilized glucan unaccounted}} = \frac{{\text{CBP glucan solubilization}}-{\text{CBP metabolites production (\% of initial glucan accounted for metabolites)}}}{\text{CBP glucan solubilization}} \times 100 \end{aligned}$$

## Additional files

**Additional file 1: Fig. S1.** Enzymatic hydrolysis (EH) glucan yield time profile on autoclaved vs. unautoclaved switchgrass with (a) 15 mg protein/g glucan and (b) 65 mg protein/g glucan enzyme loadings of Accellerase^®^ 1500. A 0.5 wt% glucan loading with a working mass of 50 g was used for EH which was performed in triplicates with controls at 50 °C, 150 rpm.

**Additional file 2: Fig. S2.** *C. thermocellum* growth profile in terms of production of metabolites (ethanol + acetic acid + lactic acid) and pellet nitrogen content (g/L) as a proxy for cell growth for 24 h under growth conditions in a bottle with 200 mL working volume and 5 g/L glucan Avicel^®^ PH-101 loading without active pH control in MOPS buffer. Measured in triplicates from one culture bottle.

## Abbreviations

CBP: consolidated bioprocessing
MTC: media for thermophilic clostridia
CELF: co-solvent-enhanced lignocellulosic fractionation
DA: dilute acid pretreatment
Alk: dilute alkali pretreatment
HT: hydrothermal pretreatment
ATP: adenosine triphosphate
THF: tetrahydrofuran
