# Supplementary material for: Biomass augmentation through thermochemical pretreatments greatly enhances digestion of switchgrass by Clostridium thermocellum
Source: Biotechnol Biofuels. 2018 Aug 4;11:219. doi: 10.1186/s13068-018-1216-7 (PMC6076393; doi:10.1186/s13068-018-1216-7)
Supplement: Supplementary file 1 — Additional file 1: Fig. S1. Enzymatic hydrolysis (EH) glucan yield time profile on autoclaved vs. unautoclaved switchgrass with (a) 15 mg protein/g glucan and (b) 65 mg protein/g glucan enzyme loadings of Accellerase® 1500. A 0.5 wt% glucan loading with a working mass of 50 g was used for EH which was performed in triplicates with controls at 50 °C, 150 rpm. [file 13068_2018_1216_MOESM1_ESM.pdf]

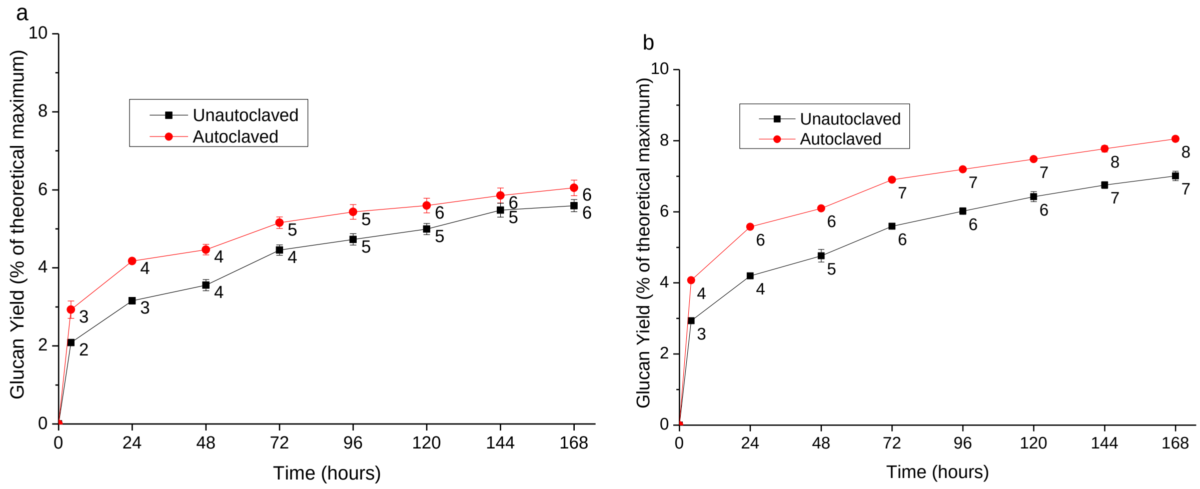

Fig. S1. Enzymatic hydrolysis (EH) glucan yield time profile on autoclaved vs. unautoclaved switchgrass with **(a)** 15 mg protein / g glucan and **(b)** 65 mg protein / g glucan enzyme loadings of Accellerase® 1500. A 5 g/L glucan loading with a working mass of 50 g was used for EH which was performed in triplicates with controls at 50°C, 150 rpm.
