# Supplementary material for: Biomass augmentation through thermochemical pretreatments greatly enhances digestion of switchgrass by Clostridium thermocellum
Source: Biotechnol Biofuels. 2018 Aug 4;11:219. doi: 10.1186/s13068-018-1216-7 (PMC6076393; doi:10.1186/s13068-018-1216-7)
Supplement: Supplementary file 2 — Additional file 2: Fig. S2. C. thermocellum growth profile in terms of production of metabolites (ethanol + acetic acid + lactic acid) and pellet nitrogen content (g/L) as a proxy for cell growth for 24 h under growth conditions in a bottle with 200 mL working volume and 5 g/L glucan Avicel® PH-101 loading without active pH control in MOPS buffer. Measured in triplicates from one culture bottle. [file 13068_2018_1216_MOESM2_ESM.pdf]

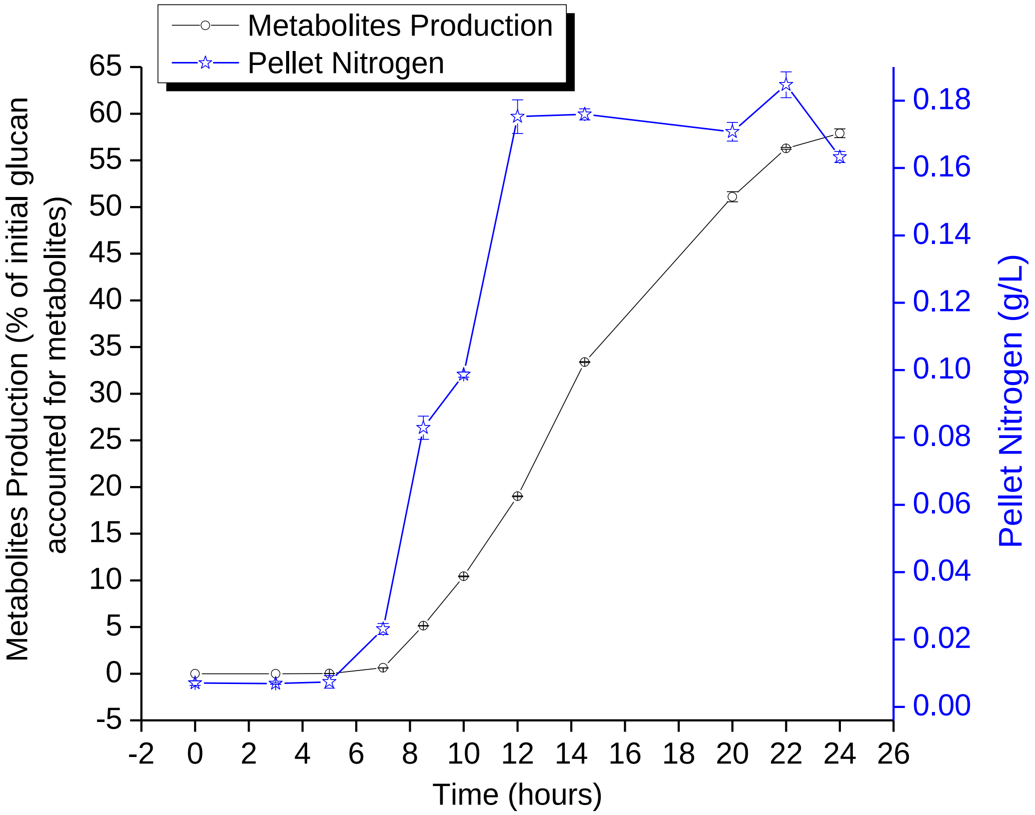

Fig. S2. *C. thermocellum* growth profile in terms production of metabolites (ethanol + acetic acid + lactic acid) and pellet nitrogen content (g/L) as a proxy for cell growth for 24 hours under growth conditions in a bottle with 200 mL working volume and 5 g/L glucan Avicel® PH-101 loading without active pH control in MOPS buffer

Measured in triplicates from one culture bottle
